# Predictors of alcohol consumption among adolescents and young adults in Lusaka, Zambia

**DOI:** 10.4314/ahs.v22i4.77

**Published:** 2022-12

**Authors:** Kasoka Mungandi, Rosemary Ndonyo Likwa, Twaambo Euphemia Hamoonga, Jerry Banda, Cosmas Zyambo

**Affiliations:** 1 Department of Population Studies & Global Health, School of Public Health, University of Zambia, P.O.Box 51110, Lusaka, Zambia; 2 Department of Epidemiology & Biostatistics, School of Public Health, University of Zambia, P.O.Box 51110, Lusaka, Zambia; 3 Department of Community and Family Medicine, School of Public Health, University of Zambia, P.O.Box 51110, Lusaka, Zambia

**Keywords:** Alcohol, adolescents, young adults, predictors, Zambia

## Abstract

**Background:**

Alcohol consumption among young people in schools and communities presents a major problem of public health concern. We determined the predictors of alcohol consumption among adolescents and young adults in Lusaka, Zambia.

**Methods:**

A cross-sectional study design was adopted. A total of 196 participants took part in the quantitative study. For the qualitative part, there were 13 participants. The study used multistage and purposive sampling methods. A semi-structured questionnaire and in-depth interviews were used. Quantitative data were analysed using STATA version 14. Ordered logistic regression analysis was used to assess the actual predictors, with confidence interval set at 95% and p-value at 0.05. Qualitative data were analysed thematically.

**Results:**

The older age category (20–24) had a greater prevalence of alcohol consumption (63.3%) than the younger age category (36.7%). Age, being employed, unconducive learning environment, limited recreation and sports activities, and adult alcohol drinking culture decreased the odds of consuming alcohol. Limited parental care support increased the odds of alcohol consumption [AOR= 4.21; 95% CI: 1.32–13.45, p=0.015]. Futile alcohol regulatory measures were cited to be contributing to alcohol consumption.

**Conclusion:**

Alcohol consumption was highly prevalent among young adults aged 20–24 years. There is need for continuous sensitization on substance abuse and its adverse effects in schools and communities at large. The strengthening, reviewing and amendment of the alcohol regulatory measures and policies should be considered.

## Background

Globally, alcohol consumption is a pervasive problem among young people below the age of 25 years^1^,^2^,^3^,^4^. The United Nations Office on Drugs and Crime (UNODC) reports that alcohol consumption continues to jeopardize the health and welfare of young people throughout the world and represents a clear threat to economic and social development^2^. The World Health Organization (WHO) also observes that alcohol consumption is a growing public health problem globally. According to the WHO estimates, 43% of the world's population aged 15 years or older have ever consumed alcohol^4^. In Africa, about 32.2% of those aged 15 years and above (21.4% aged 15–19 and 34.1% aged 20–24) have ever consumed alcohol^4^,^5^. Alcohol consumption impacts not only health but also the socio-economic sphere 5, 6. Research suggests that alcohol consumption results into problems such as HIV infection, crime and change in personality, poor academic performance, high mortality and morbidity due to injuries and road accidents^6^,^7^. Some studies in Africa have found an association between alcohol consumption and male gender, peer pressure, family history of alcohol abuse, unstable employment, poor social and coping skills, increased alcohol availability and positive expectations regarding alcohol use^5^,^8^.

In Zambia, alcohol consumption is recognized as a growing public health concern among young people^9^,^10^,^11^. Since the mid-1990s, alcohol consumption prevention interventions have been intensified in schools and communities so as to bring about increased knowledge, anti-drug attitudes and behaviour change among young people^7^. Regardless of these considerable efforts, the number of young people aged 15–24 years reported to be consuming alcohol has steadily been increasing^7^,^10^. The World Health Organization estimated the percentage of current drinkers below the age of 25 years in Zambia at 44% in 2014, and at 53% among those aged 15–19 years in 2018^4^,^5^. Alcohol consumption prevention is critical to the sustainability of not only a good public health system but also socio-economic development of the country 7,12. This study aimed at determining the predictors of alcohol consumption among adolescents and young adults in Lusaka, Zambia.

## Methods

### Study Design and Setting

A cross-sectional study design using a concurrent mixed-methods approach was employed to determine the predictors of alcohol consumption among adolescents and young adults in Lusaka, Zambia. The qualitative component was conducted in order to compliment the major quantitative component of the study. The study was conducted in two of Lusaka's high-density areas (Kanyama and Matero compound). The two compounds are situated about 6 kilometres away from the Lusaka Town Centre.

### Study Participants

The study participants comprised adolescents and young adults aged 15 to 24 years who were either resident or schooling in Kanyama and Matero compounds. The study participants were required to have been schooling and staying in the study areas for not less than two years preceding the study. The study also included key informants considered to be policy makers/implementers and community representatives. Among them included the Civic Leaders, Drug Enforcement Commission (DEC) Officers and Parents.

### Study Sampling

The study used multi-stage sampling for the quantitative component. Firstly, the two strata (Kanyama and Matero) were purposively selected because of being densely populated with high prevalence of risky behavioural activities among young people. Simple random sampling was applied to select one public secondary school from each stratum. Pupils aged 15 to 19 years who were in their 9^th^, 11^th^, and 12^th^ grades were selected as study participants. Pupils in Grade 8 and Grade 10 were excluded as most of them wee relatively new at the selected schools. Simple random sampling was used to select eligible pupils using class registers. Young adults aged 20–24 years who were patronizing the bars in the communities were selected using systematic random sampling. The first participant was selected randomly, thereafter, a 30-minute interval was used to select the subsequent participants at each drinking place.

The qualitative component comprised a total of 13 key informants, with a distribution of 2 Civic Leaders, 1 DEC officer and 10 parents or guardians selected conveniently for in-depth interviews. We had anticipated that 20 key informants would be enough to gain information sought. However, only 13 key informants were interviewed as information saturation was met.

### Variables and Measurements

The dependent variable for this study was the prevalence of alcohol consumption which was measured by the number of young people consuming alcohol in relation to the total sample responding to the questions relating to: “how often did you take a drink containing alcohol in the past 30 days?” Those who answered ‘YES’ to the question “have you ever taken a drink containing alcohol in the past 30 days?” were considered as consumers of alcohol and those who answered ‘NO’ as non-drinkers. The independent variables were Socio-demographic characteristics: age (age at last birthday in years); sex (classified as male or female); education (none, primary, secondary or tertiary); including economic characteristics such as occupation (student, employed, self-employed or unemployed); adult alcohol drinking culture (low or high prevalence of adult alcohol consumption in the communities); peer pressure (based on productive or non-productive peer groups); recreation and sports activities (supportive or non-supportive in the community); alcohol control measures (effectively or non-effectively utilized in the community); parental alcohol drinking culture (defined as uncontrollable drinking of alcohol among parents); limited parental care support (measured as adequate or inadequate parental support over their children); school learning environment (conducive or unconducive for behaviour change); and easy access to alcohol pubs facilities (based on presence or absence of liquor outlets in the communities).

### Data Collection Method

Quantitative data was obtained through self-administered semi-structured questionnaires. The questionnaire consisted of 2 parts. The first part included questions on the general characteristics of the participants, such as age, sex, education and occupation. The second part consisted of questions regarding alcohol consumption, such as “have you ever taken a drink containing alcohol in the past 30 days?” and “how often did you take a drink containing alcohol in the past 30 days?” All completed questionnaires were kept in a secured location before they were coded with a number and entered into excel for cleaning.

For the qualitative part, in-depth interviews were conducted to collect additional data for the study. The key informants were contacted individually and asked to participate in the study. Both face-to-face and phone interviews that were audio-recorded were conducted with consenting participants.

### Statistical Analysis

Descriptive statistics were used to summarize the characteristics of the participants, including frequencies and proportions for categorical data and means and standard deviations for continuous data. Characteristics were compared between those who did/did drink using the Chi-square test. Fisher's exact test was used for variables with one of the frequencies less than 5. Univariate ordered logistic regression analysis was used to determine associations between alcohol consumption and independent vari. The variables that showed a statistically significant association with alcohol consumption at 10% significance cut-off point were fitted into the multivariate ordered logistic regression analysis in order to obtain the adjusted odds ratios. A p-value less than 0.05 was considered significant with an associated 95% confidence interval in the multivariate ordered logistic regression analysis. STATA (Version 14, college station, Texas USA) was used for data analysis.

Thematic analysis, which is a way of identifying common themes within the data in order to be grouped in a clear and organized manner^13^, was employed. Data obtained from the in-depth interviews with key informants was transcribed verbatim and coded manually. Coded reports were further generated and analysed in relation to the objectives of the study.

### Ethical Considerations

The study received ethical approval from the University of Zambia Biomedical Research Ethics Committee (UNZABREC) and National Health Research Authority (NHRA). Related approvals were also sought from the Lusaka City Council, Drug Enforcement Commission and Lusaka District Education Board Secretary (DEBS) on behalf of the Ministry of General Education. Written consents were obtained from all participants aged 19–24 years prior to data collection in schools and communities. Additionally, written assents were obtained from the school managers on behalf of the parents to obtain data from pupils below 18 years. Pupils below 18 years were also well informed about the study prior to data collection.

## Results

The quantitative component comprised of 196 participants (49% aged 15–19 years and 51% aged 20–24 years) with the mean age of 20 (3 SD). About 52.6% of the respondents were males and (47.4%) females. A total of 80.6% of those aged 15–24 years (36.7% aged 15–19 and 63.3% aged 20–24) ever consumed alcohol (Table 1). A total of 30.6% reported having consumed alcohol weekly followed by those who consumed daily (27%) and monthly (23%). More respondents (39.3%) reported having consumed six bottles of alcohol per day (Table 1).

**Table 1:** Socio-demographic characteristics of the study participants according to alcohol consumption

	Alcohol Consumption		P-value
Characteristic	Yes= 158	No= 38	
**Age**			
15–19 years	58 (60.40%)	38 (39.60%)	0.001
20–24 years	100 (100.0%)	0 (0.0%)	
Total	158 (80.61)	38 (19.39%)	
**Sex**			
Female	67 (72.04%)	26 (27.96%)	0.004
Male	91 (88.25%)	12 (11.65%)	
Total	158 (80.61%)	38 (19.39%)	
**Frequency of Alcohol Consumption per Period**			
Never	0 (0.0%)	38 (100%)	0.001
Daily	53 (100.0%)	0 (0.0%)	
Weekly	60 (100.0%)	0 (0.0%)	
Monthly	45 (100.0%)	0 (0.0%)	
Total	158 (80.61%)	38 (19.39%)	
**Alcohol Consumption in Millilitres per Day**			
None	0 (0.0%)	38 (100%)	0.001
375 ML to 1125 ML (3 Bottles)	58 (100.0%)	0 (0.0%)	
1125ML to 1875 ML (6 Bottles)	77 (100.0%)	0 (0.0%)	
1875 ML to 2625 ML (8 Bottles)	23 (100.0%)	0 (0.0%)	
Total	158 (80.61%)	38 (19.39%)	
**Location of Alcohol Pubs Facilities**			
Closer to Households	131 (83.97%)	25 (16.03%)	0.019
Far from Households	27 (67.50%)	13 (32.50%)	
Total	158 (80.61%)	38 (19.39%)	
**Alcohol Regulatory Measures (10hours to** **24hours**			
Adherence to Opening/Closing Hours	77 (72.64%)	29 (27.36%)	0.002
No adherence to Opening/Closing Hours	81 (90.0%)	9 (10.0%)	
Total	158 (80.61%)	38 (19.39%)	
**Peer Pressure**			
Yes	155 (82.01%)	34 (17.99%)	0.010
No	3 (57.14%)	4 (42.86%)	
Total	158 (80.61%)	38 (19.39%)	
**Easy Access to Alcohol**			
Yes	151 (84.36%)	28 (15.64%)	0.001
No	7 (41.18%)	10 (59.82%)	
Total	158 (80.61%)	38 (19.39%)	
**Unemployment**			
Yes	145 (84.30%)	27 (15.70%)	0.001
No	13 (54.17%)	11 (45.83%)	
Total	158 (80.61%)	38 (19.39%)	
**Alcohol Education**			
Yes	147 (86.47%)	23 (13.53%)	0.001
No	11 (42.31%)	15 (57.69%)	
Total	158 (80.61%)	38 (19.39%)	
**Parental Drinking Pattern**			
Yes	141 (88.10%)	19 (11.90%)	0.001
No	17 (47.22%)	19 (52.78%)	
Total	158 (80.61%)	38 (19.39%)	

**Figure 1:**
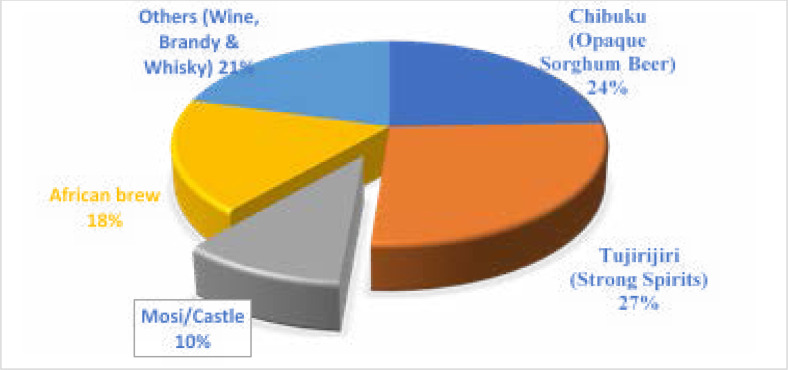
Types of alcohol ever available in the community

**Figure 2:**
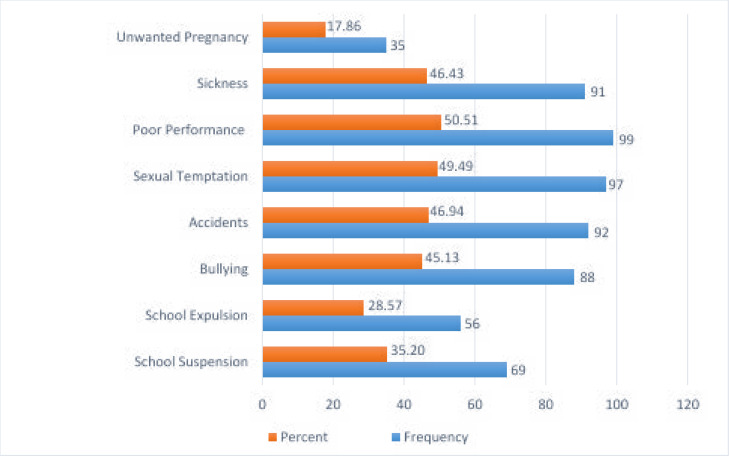
Effects of alcohol consumption among adolescents and young adults

Overall, 27% of the study participants reported Tujirijiri (strong spirits) to be a common type of alcohol ever consumed in the community, followed by Chibuku (Opaque Sorghum Beer) (24%) (Figure 1). Half of the participants reported that alcohol consumption contributed to poor academic performance and sexual temptation among young people and some had experienced accidents (46.9%), sickness (46.4%), and bullying (45.1%) (Figure 2).

In the univariate ordered logistic regression analysis, those aged 20–24 years, being employed, unemployed, peer pressure, easy access to alcohol, unconducive school learning environment, parental alcohol drinking culture, limited recreation/sports activities, limited parental care support, futile alcohol control measures and adult alcohol drinking culture were strongly associated with alcohol consumption. Females were twice as likely to consume alcohol compared to males while having attained secondary education increased the odds of consuming alcohol 11-fold (Table 2).

**Table 2:** Predictors of alcohol consumption among Adolescents and Young Adults in Lusaka Zambia

Predictor	UOR (95%CI)	P-value	AOR (95% CI)	P-value
**Age**				
15–19 years	1.00		1.00	
20–24 years	0.65 (0.59, 0.71)	0.001	0.77 (0.68, 0.86)	0.001^a^
**Sex**				
Male	1.00		1.00	
Female	2.54 (1.51, 4.26)	0.001	1.59 (0.87, 2.91)	0.132
**Marital Status**				
Single	1.00		1.00	
Engaged	0.60 (0.15, 2.46)	0.483	0.68 (0.13, 3.38)	0.633
**Education**				
Primary	1.00		1.00	
Secondary	11.2 (3.23, 38.82)	0.001	1.89 (0.49, 7.37)	0.358
Tertiary	1.59 (0.43, 5.96)	0.489	1.62 (0.40, 6.55)	0.501
**Occupation**				
Student	1.00		1.00	
Employed	0.04 (0.02, 0.11)	0.001	0.33 (0.12, 0.96)	0.042^a^
Unemployed	0.08 (0.04, 0.16)	0.001	0.54 (0.22, 1.37)	0.198
**Peer pressure**				
No	1.00		1.00	
Yes	0.15 (0.03, 0.62)	0.009	0.85 (0.13, 5.57)	0.862
**Easy access to alcohol facility**				
No	1.00		1.00	
Yes	0.19 (0.04, 0.32)	0.001	1.23 (0.36, 4.22)	0.744
**School environmental Climate**				
No	1.00		1.00	
Yes	0.08 (0.04, 0.15)	0.001	0.36 (0.16, 0.80)	0.012a
**Parental drinking pattern**				
No	1.00		1.00
Yes	0.11 (0.05, 0.22)	0.001	0.49 (0.18, 1.36)	0.171
**Recreation & sports activities**				
No	1.00		1.00	
Yes	0.08 (0.03, 0.16)	0.001	0.37 (0.14, 1.01)	0.050a
**Parental care support**				
No	1.00			
Yes	0.17 (0.08, 0.37)	0.001	4.21 (1.32, 13.45)	0.015^a^
**Alcohol Control measures**				
No	1.00		1.00	
Yes	0.06 (0.03, 0.12)	0.001	0.49 (0.17, 1.42)	0.187
**Adult drinking pattern**				
No	1.00		1.00	
Yes	0.08 (0.04, 0.16)	0.001	0.31 (0.12, 0.82)	0.017^a^

a significant level= 0.05.

In the multiple ordered logistic regression analysis, age (20–24) was 23% less likely to influence alcohol consumption among young people (AOR= 0.77; 95% CI: 0.68–0.86, p<0.001). Results also show that unconducive school learning environment was 64% less likely to influence the consumption of alcohol (AOR= 0.36; 95% CI: 0.16–0.80, p=0.012). Being employed was 67% less likely to influence alcohol consumption (AOR= 0.33; 95% CI: 0.12–0.96, p=0.042). Furthermore, adult alcohol drinking behavior decreased the chances of consuming alcohol by 69% among young people (AOR= 0.31; 95% CI: 0.12–0.82, p=0.017). On the other hand, limited parental care support increased the odds of alcohol consumption (AOR= 4.21; 95%CI: 1.32–13.45, p=0.015) (Table 2).

The qualitative results are presented in three parts: firstly, the factors associated with alcohol consumption; secondly, the potential impacts of alcohol consumption and thirdly, the availability of alcohol policy guidelines (Table 3).

**Table 3:** Key Thematic Categories

Main Theme	Sub-Theme
Factors associated with alcohol consumption	Peer pressure
	Lack of employment
	Experimentation and fun
	Ignorance
	Lack of parental care support
	Easy accessibility and cheap alcohol
	Fragile alcohol regulatory measures
Potential Impacts of Alcohol Consumption	HIV/AIDS
	Death/injuries
	Poor academic performance
	Anti-social behaviours
Availability of Alcohol Policy Guidelines	Weak and inadequately followed

### Factors Associated with Alcohol Consumption

Study participants acknowledged that the level of alcohol consumption among adolescents and young adults was very high in Lusaka. The participants further cited many factors associated with alcohol consumption among young people. These factors were related to individual, interpersonal, household, community, societal and institutional factors. More than half of the participants acknowledged some individual and interpersonal behavioural factors that included group influence, unemployment, experimenting, boredom and rebellion, among others to contribute to the existing levels of alcohol consumption among young (15–24). One community representative stated:

*There is a high number of young people taking alcohol in this township and the major cause is failure to find what to do or lack of employment influence them to consume alcohol as a way of keeping themselves busy (KI #1, Parent).*

### Additional representatives had this to say:

*We are not happy with the way younger boys and girls are drinking beer in nowadays. If were to visit any bar in this community there are only small-small girls and boys taking these so called tujirijiri. This is attributed to group influence as parents are failing to guide and raise their children properly. The life style, experimenting, boredom and rebellion also commonly entice young people to consume alcohol (KI #2 & 6, Parents).*

The participants concluded that the number of young people consuming alcohol was increasing due to the challenges faced in their homes and the community at large. The common challenges stated included poverty, easy accessibility and cheap alcohol, lack of parental guidance and parental alcohol drinking pattern, as noted by one policy implementer:

*Honestly speaking the parents are contributing to this vice among the younger youth especially those who engage in drinking of beer. Parents need to be role models to their children failure to which they will end up consuming alcohol (KI #13, DEC).*

### Another parent added that:

*Alcohol has become so cheap today as compared to the past. This generation is different from the old one because what is happening to the young ones today is scary and this requires serious interventions from their parents and the government (KI #7, Parent).*

The societal status and institutions at large were cited to be the main contributing factors related to alcohol consumption among the young people. The participants highlighted that there were no regulatory control measures as depicted in the following statement:

*There is poor application of alcohol guidelines in our township and this has led to availability of alcohol pubs near homes resulting in not enjoying our sleep (KI #9, Parent).*

**On the other hand, one civic leader maintained:**

*The law is known among the bar owners. Although those selling alcohol behave like they were from the same mothers and others do not have liquor licenses. It is really up to the government to ensure that the closing and opening of bars in markets and the community at large is followed as they are becoming unruly (KI #11, Civic Leader).*

### Potential Impact of Alcohol Consumption

When the participants were asked on the possible problems that could result from alcohol consumption among adolescents and young adults, the majority reported many problems associated with alcohol consumption. These problems were related to socioeconomic and public health impacts. The participants perceived that regular intake of alcohol among the young people is harmful or dangerous as it could result into several health problems. Some of the participants further stated alcohol consumption to be beneficial when taken in moderation as one DEC officer indicated:

*Males that drink alcohol in moderation such as red wine, diminishes their risk of developing prostate cancer. However, excessive drinking can result in the damage of the brain cells permanently, stroke, heart attack, infertility, impotence, weak body muscles, addiction and liver damage (KI #13, DEC)*.

**Some participants further claimed that:**

*We are not happy with this alarming situation as it impacts them negatively by getting diseases such as HIV/AIDS and having children without their fathers (KI #2, Parent). There is also death or killings and injuries involved among boys and girls. For example, there is one boy who was killed recently in Mtendedere compound by another boy over a simple quarrel after drinking alcohol (KI #2 & 12, Parent and Civic leader)*.

The majority of the participants revealed that alcohol consumption among the younger people may result into anti-social behaviours such as theft, domestic violence, prostitution, child abuse, disrespect and fighting, and poor academic performance, poor national development and financial difficulties. One parent said:

*Excessive intake of alcohol destroys people's future by reducing on their academic performance and this weakens national development as the younger youths are the backbones or engine for every society to move forward (KI #9, Parent)*.

**Additionally, some participants also affirmed:**

*In the past, we had younger youths taking alcohol, but these days it is different when they get drunk because they insult elders and become free to talk anyhow because alcohol percentages are very high. Alcohol intake has further led to high levels of child abuse such as defilement and theft, and someone normal can't do that. As parents we are also suffering every day and night as we are being insulted, disrespected and beaten at times (KI #4 & 5, Parents).*

### Availability of Alcohol Regulatory Policies

Zambia has got laws and by-laws such as the Liquor Licensing Act of 2011 and the National Alcohol Policy of 2018 meant to regulate the production, distribution, selling and buying, and to some extent consumption of alcohol. Regardless of that considerable effort, the majority of the participants reported that alcohol policy guidelines were not adequately followed. Despite some participants reporting that alcohol policy guidelines were adequately followed, the majority did not know about the existence of alcohol policy guidelines, as one parent stated:

*We had strong alcohol regulations in Zambia not these days, they are weak. We don't even know if they exist because everyone is free to do whatever they want in Zambia without facing any courts of law. Bar owners also don't care whoever takes their beer as what they want is money (KI #10, Parent)*.

## Discussion

Findings from this current study reveal that the prevalence of alcohol consumption was very high (63.3%) among the older participants (20–24) compared to those aged 15–19 years (36.7%). This finding is contrary to findings from a study in Northern Thailand in which two-thirds of adolescents aged 15–19 years had a higher prevalence (61.5%) of alcohol consumption than those aged 20–24 years^14^. Similarly, in other studies in Zambia, the estimated prevalence of alcohol consumption among adolescents was 53% and 43.7% respectively^5^,^15^. This could suggest that adolescence is a genesis phase of alcohol intake in the communities. A study in Western Australia showed that alcohol initiation usually occurs in early adolescence with an increase in consumption throughout adolescence and early adulthood followed by gradual decrease over the following years^16^.

Findings further revealed that most participants consumed alcohol weekly, followed by those who consumed daily and monthly. According to Zverev^17^, excess alcohol use among young people clearly shows a possibility of alcohol dependence or disorders in the future. The study also discovered a higher proportion of young people having ever consumed *tujirijiri* (strong spirits), *chibuku* (opaque sorghum beer) and other alcohol types. This is comparable to another study conducted in Bhaktapur where most of the respondents (44.7%) drank beer and other related types of beer^18^, and young people favoured beer and local whisky because of their easy accessibility and affordability in the communities^14^.

The study found an association between those aged 20–24 years and alcohol consumption. This correlation is consistent with other studies which reported that age is a significant predictor of alcohol consumption among young adults aged 20–24 years (p=0.031) and that alcohol consumption increased with age among the young people^19^, ^20^, ^21^. On the contrary, the in-depth interviews for this study, showed that community bars were ever filled with adolescents consuming tujirijiri (strong spirits) due to group influence, poor parental guidance and easy access to alcohol.

In 2019, a study found that young adults with employment had greater odds of engaging in alcohol use compared to those without employment^14^. Another study in nine ASEAN countries also found that having an income was a significant factor for alcohol use among the younger people^22^. A study conducted in ten different European countries found similar results showing that income was associated with alcohol use among younger people^23^. In our study, we found that being employed was significantly associated with a decreased chance of consuming alcohol as compared to those schooling and unemployed. Similarly, qualitative results of the present study found that majority of young adults engaged in alcohol consumption due to failure to find what to do or lack of employment as a way of keeping themselves busy.

Our study found that unconducive school learning environment could be a possible explanation for the existing levels of alcohol consumption among adolescents. In Botswana, a study found that adolescents who experienced stresses in the family, peer relationship and schools were at risk for alcohol and drug use as compared to those with support from peers, family, school and religiosity^24^. Among Kenyan university students a study linked alcohol consumption to community factors such as availability of alcohol, low prices, density distribution of outlets, social settings and campus customs as most institutional campuses have bars that sell alcohol 25. Participants from key informant interviews cited rebellion, curiosity and ignorance as factors influencing adolescents to consume alcohol.

We found not engaging in recreation and sports activities to be strongly associated with alcohol consumption among young people. In 2014, Habulembe^10^ found sports activities in Zambia to be a challenge because of lack of facilities and motivation, hence making young people to find it pointless to indulge into various sports activities as compared to consuming alcohol. The in-school adolescents and young adults in the communities need to be empowered with co-curricular skills and knowledge in order to avoid such negative health risk behaviours^26^.

Our study found alcohol consumption to be related to poor academic performance, sexual temptation, bullying, sickness, accidents and unwanted pregnancies. Kubwalo and others^27^ further confirmed that Zambian young people engaged in fights with their friends under the influence of alcohol, and expulsion, suspension, disrespecting teachers and absenteeism among the in-school adolescents and young adults were the major concern due to excess alcohol consumption. This could be a sign that alcohol consumption among young people is dire and failure to instil alcohol discipline would ruin their future. Pacoricona et al. reported that parents need to be good role models to their children, failure to which they will end up consuming alcohol^28^. Their study further concluded that divorced/separated parents strongly influenced alcohol use among young people^28^. This present study found limited parental care support to be four times as likely to influence alcohol consumption among young people. This is parallel to qualitative results that revealed that parents were the major contributors of adolescent alcohol use in the communities especially those who consumed beer.

A study done by Yeide found that if alcohol use is an integrated part of the culture, the younger people may be also more likely to engage in alcohol consumption^29^. This is in agreement with our study in which the attitudes of adults towards alcohol drinking in communities was found significantly associated with alcohol consumption among adolescents and young adults. Fuhr and Gmel^30^ also reported that high levels of alcohol consumption among younger people is a true reflection of national adult drinking culture. It is important to note that increased rates of adult alcohol drinking culture contribute to a high prevalence of alcohol consumption among young people in the communities by making them vulnerable to learning the adult alcohol drinking behaviour and lead a life full of experimentation and fun. This was supported by the in-depth interview results for this study, where the participants viewed the current generation to be different from the old generation due to some learnt behaviours, experiments and changes in their life styles.

Brand and others^31^ found that alcohol control policies such as restrictions on advertising and availability of alcohol reduced alcohol consumption among the young people. In like manner, Zambia has statutory laws and by-laws aimed at regulating the production, distribution, selling and buying, and to some extent consumption of alcohol and among these include the Liquor Licensing Act of 2011 and the National Alcohol Policy of 2018. Despite the availability of such laws, the in-depth interviews for this study revealed non-adherence of alcohol regulatory measures in the communities in relation to opening-closing hours and easy accessibility. Masiye 7 further observed that regulations on alcohol consumption, specifically those aimed at ensuring that no underage is allowed to enter or purchase alcohol in the communities were weak and fragile.

## Limitations

The study had few limitations. Firstly, due to the sensitive nature of the topic, it is possible that some respondents may have felt uncomfortable and insecure to respond to all questions fully and freely, thereby resulting in information bias. Secondly, the findings may not be generalized to Zambia as a whole since the study was conducted only in two high density areas of Lusaka, without representation from other provinces. Despite these limitations, the study was able to highlight adolescent and young adults' behaviour apropos alcohol consumption, including factors associated with it. This information is useful to policy makers and for Public Health programming, specifically interventions aimed at discouraging young people from engaging in risky behaviours such as excessive alcohol consumption.

## Conclusion

Alcohol consumption is a growing public health concern among young people in Kanyama and Matero. Our study revealed that the prevalence of alcohol consumption is very high among young adults than adolescents. The factors include: those aged 20–24 years, occupation, unconducive school learning environment, limited recreation and sports activities, limited parental care support, and adult alcohol drinking culture. The laws and policies in place to control alcohol consumption should be appropriately reviewed, implemented and enforced. There is need also for continued alcohol education and training in schools, alongside the use of mass media platforms for community-based education awareness programs on the health risks of alcohol consumption among young people. Strengthening of the monitoring systems in the communities should further be considered as a possible measure for reducing the alcohol-related health risks on this vulnerable population in our communities.
